# Does aerial baiting for controlling feral cats in a heterogeneous landscape confer benefits to a threatened native meso-predator?

**DOI:** 10.1371/journal.pone.0251304

**Published:** 2021-05-07

**Authors:** Russell Palmer, Hannah Anderson, Brooke Richards, Michael D. Craig, Lesley Gibson

**Affiliations:** 1 Biodiversity and Conservation Science, Department of Biodiversity, Conservation and Attractions, Western Australia, Australia; 2 School of Biological Sciences, University of Western Australia, Western Australia, Australia; 3 Environmental and Conservation Sciences, Murdoch University, Western Australia, Australia; University of Southern Queensland, AUSTRALIA

## Abstract

Introduced mammalian predators can have devastating impacts on recipient ecosystems and disrupt native predator–prey relationships. Feral cats (*Felis catus*) have been implicated in the decline and extinction of many Australian native species and developing effective and affordable methods to control them is a national priority. While there has been considerable progress in the lethal control of feral cats, effective management at landscape scales has proved challenging. Justification of the allocation of resources to feral cat control programs requires demonstration of the conservation benefit baiting provides to native species susceptible to cat predation. Here, we examined the effectiveness of a landscape-scale *Eradicat®* baiting program to protect threatened northern quolls (*Dasyurus hallucatus*) from feral cat predation in a heterogeneous rocky landscape in the Pilbara region of Western Australia. We used camera traps and GPS collars fitted to feral cats to monitor changes in activity patterns of feral cats and northern quolls at a baited treatment site and unbaited reference site over four years. Feral cat populations appeared to be naturally sparse in our study area, and camera trap monitoring showed no significant effect of baiting on cat detections. However, mortality rates of collared feral cats ranged from 18–33% after baiting, indicating that the program was reducing cat numbers. Our study demonstrated that feral cat baiting had a positive effect on northern quoll populations, with evidence of range expansion at the treatment site. We suggest that the rugged rocky habitat preferred by northern quolls in the Pilbara buffered them to some extent from feral cat predation, and baiting was sufficient to demonstrate a positive effect in this relatively short-term project. A more strategic approach to feral cat management is likely to be required in the longer-term to maximise the efficacy of control programs and thereby improve the conservation outlook for susceptible threatened fauna.

## Introduction

The introduction of mammalian predators outside their natural range can have severe consequences on recipient ecosystems [1–3] and be particularly disruptive to native predator–prey relationships [4, 5]. Introduced predators tend to adapt well to their new environment [2], and native species become ‘easy prey’ probably due to behavioural naïveté to these novel predators [6, 7]. Moreover, once predators have established in an area, effectively controlling them poses a significant challenge [8, 9]. The detrimental impacts of introduced predators on native biodiversity can be seen in countries like Australia and New Zealand with disproportionately more extinctions of native species (mammals–Australia; birds–New Zealand) than other developed countries [2, 10, 11]. In Australia, predation by the introduced red fox (*Vulpes vulpes*) and feral cat (*Felis catus*) has been implicated in the decline and extinction of many native species [11–13]. Large scale fox-baiting programs have been shown to be relatively effective in reducing their impact on native fauna [14, 15]. However, there is also some evidence that feral cat populations can increase in response to fox control [16, 17].

To avert further extinctions of native fauna from the Australian mainland and to improve their prospects of recovery, land managers need effective and affordable methods to control the detrimental impacts caused by feral cats [12, 18]. There have been considerable advances made in the lethal control of feral cats in the past decade [19–21], as evidenced by the recent removal of this invasive predator from Dirk Hartog Island off Western Australia (WA). At 630 km^2^, Dirk Hartog Island is the largest successful feral cat eradication campaign for an island in the world to date [22]. Controlling feral cats in open mainland systems, however, presents an ongoing and formidable challenge to conservation practitioners. Local removal of feral cats from the landscape effectively forms a ‘sink’, creating opportunity for other cats to either encroach as territory becomes available or locally reproduce and occupy the space [23, 24]. Therefore, control efforts should be continual, or relatively consistent, and large-scale, for any benefit to native fauna species to be realised–a costly exercise and a challenge in itself [8, 25].

The aerial application of toxic baits is currently considered to be the most effective and efficient method for controlling feral cats at a large scale, if the risk to non-target species is minimal [20, 26–28]. The recent registration of the *Eradicat®* feral cat bait (i.e., sausage-style baits containing the toxin sodium fluoroacetate or 1080) may provide land managers with an affordable tool to control feral cats at the scale required [20, 28]. Sodium fluoroacetate is a naturally occurring plant toxin that is found in more than 30 species of mainly Western Australian plants [29]. Owing to their long evolutionary exposure to these plants, many Australian animal species show a higher degree of tolerance to the toxin than most unadapted non-native species, particularly eutherian carnivores [29, 30]. However, the operational use of *Eradicat®* in northern Australia is currently prohibited as the risk to the endangered northern quoll (*Dasyurus hallucatus*) in the wild had not been assessed at the time of bait registration [31].

Like most arid regions of the Australian mainland, the Pilbara bioregion of northwest WA, has suffered significant biodiversity loss in the past 200 years. Twelve species of terrestrial mammal have become extinct from the mainland and another seven species have declined [32, 33]. A review of the conservation values, threats, and management options for biodiversity conservation in the Pilbara, identified feral cat control as one of the top three management strategies to be implemented based on the relatively low cost and high benefit of this option for multiple species of threatened fauna, including northern quolls [34]. However, this review also indicated that the probability of success of cat control over a 20-year management period was uncertain, with a 49% predicted chance of success [34]. This uncertainty is likely to be related to the challenge of effectively controlling feral cats in the expansive topographically complex landscapes across this region [28].

There are few published reports on the efficacy of multi-year landscape-scale feral cat baiting operations. Two exceptions are the Fortescue Marsh feral cat control program in the Pilbara, which has been ongoing since 2012 [28], and another in the Matuwa Kurrara Kurrara Indigenous Protected Area (Matuwa) in central WA commencing in 2003 [20, 27]. The first of these demonstrated a decline in site occupancy of cats in all five years of the study, although repopulation by cats each year was relatively rapid. At Matuwa, the annual baiting strategy provided sustained control of feral cats for over five years [27], but in the longer-term, cat activity returned to near pre-bait levels and prompted the use of complementary control techniques [20]. Neither study examined the response of native species to feral cat control. A further study examining the efficacy of integrating landscape-scale feral cat control into an existing predator control program on the south coast of WA reported varying success of feral cat baiting on cat populations, though native species appeared to respond positively [35]. All these studies were conducted in relatively low-relief landscapes.

In the Pilbara, recent research to assess the survivorship of northern quolls before and after a trial aerial feral cat baiting program provided evidence that the application of *Eradicat*® poses a low poison risk to northern quolls [31]. The next step was to quantify the response of both cats and northern quolls to landscape-scale feral cat baiting to demonstrate that this management action will improve the conservation outlook for northern quoll populations. The Pilbara northern quoll populations are an important stronghold for this species [36, 37], given that the invasion of the poisonous cane toad (*Rhinella marina*) elsewhere in northern Australia has seen dramatic declines in once locally abundant populations [11, 38]. Feral cats are also considered a threat to northern quolls and reducing their impact on this species is a high conservation priority [39].

Here, we assessed the response of both feral cats and northern quolls to the annual aerial application of the *Eradicat®* bait in a heterogeneous rocky landscape in the Pilbara over a four-year period by examining changes in activity patterns using camera traps, and GPS collars fitted to cats, at a treatment (baited) and reference (non-baited) site. Our overarching hypothesis was that landscape-scale baiting of feral cats would confer a benefit to northern quolls i.e., reduce predation pressure.

## Methods and materials

This study was approved by the Department of Biodiversity, Conservation and Attractions (DBCA) Animal Ethics Committee (AEC 2015/16 and 2018/04) and by annual DBCA 1080 baiting risk-assessment plans.

### Study site

**Fig 1:**
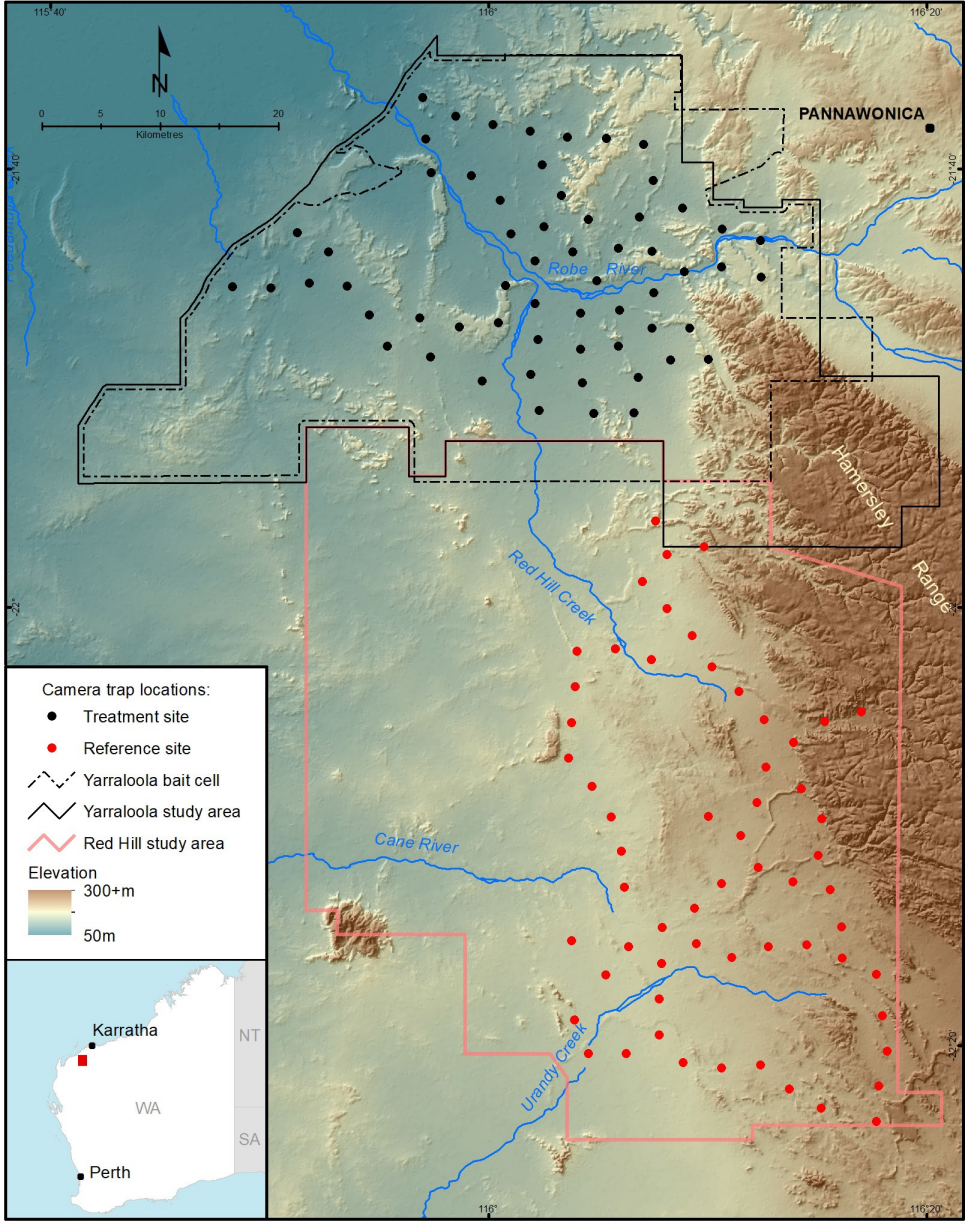
Location of the treatment (Yarraloola) and reference (Red Hill) sites and camera trap locations in the Pilbara region of Western Australia. The modified bait cell used for 2018 and 2019 trials is also shown.

The study was conducted on two adjoining cattle stations (Yarraloola 160,000 ha and Red Hill 180,000 ha) in the west Pilbara of WA (Fig 1). Yarraloola was the baited treatment site and Red Hill was the non-baited reference site. These stations were selected as they have similar topographies and land systems, and the same enterprise manages the cattle herds. Road access was extensive due to pastoral and mining operations, including exploration tracks in the rocky upland landscapes. The eastern boundaries of both stations lie along the western edge of the rugged Hamersley Range. The adjacent sections of this range (Unallocated Crown Land) were also part of the study area (Fig 1).

This area experiences a semi-arid climate typical of the Pilbara bioregion. Summers are very hot and winters mild. Rainfall is characteristically extremely variable and follows a loose bi-modal pattern with most of the rain falling during January, February and March in association with tropical cyclone and heat-trough events. A second, smaller rainfall peak occurs in May and June as a result of southern frontal systems. Average annual rainfall for Pannawonica (the nearest weather recording station, 25 km northeast of Yarraloola) and Red Hill are 407 and 363 mm, respectively [40]. Annual rainfall for Pannawonica and Red Hill were similar over the study period with 2017 being the wettest year at both stations (538 and 366 mm, respectively) followed by 2016 (391 and 346 mm) then 2018 (323 and 336 mm) and 2019 being the driest year at both stations (238 and 174 mm). The only notable difference during the study period was a tropical low that passed over Yarraloola in February 2017 delivering 319 mm to Pannawonica but only 159 mm to Red Hill [40].

The two stations have similar landforms and vegetation. The central lowland systems of the study area commonly feature stony plains and spinifex hummock grasslands with scattered low open woodlands of *Acacia* species [41]. These areas are interspersed by the Robe Pisolite [42], which form flat-topped mesas and breakaways. There are also other substantial rocky hills of various geologies, all supporting spinifex grasslands and sparse eucalypt woodlands [41]. The western side of the study area is predominately lowland plains featuring rolling stony plains and spinifex hummock grasslands, interspersed with isolated low rocky hills [41]. The area of habitat suitable (rugged rocky uplands and complex riverine systems; [37]) for northern quolls was similar at both stations, comprising 35–40% of the study sites. Given the home range size of northern quolls determined in a previous study [31], the distance between the two study locations was sufficient to ensure independence.

Prior to the commencement of this study, both stations were aerially baited each September with dried meat baits containing 6 mg of 1080 to control wild dogs (dingoes and other free-ranging dogs; *Canis familiaris*) as part of a coordinated Pilbara-wide operation. Thereafter, the primary control method used to control wild dogs was ground shooting. In the precursor to this study, a 20,000-ha area of Yarraloola was baited with *Eradicat®* in July 2015 to investigate the non-target risks on the survival of free-ranging northern quolls [31].

### Aerial baiting

Aerial baiting occurred over two days in July over the four years (26–27 July 2016; 16–17 July 2017; 9–10 July 2018; 8–9 July 2019). This scheduling coincided with the coolest period of the year in the Pilbara when the availability of alternative prey was predicted to be lowest, maximising bait uptake by feral cats [43]. Bait degradation from unseasonal rainfall, hot weather and ant attack was also likely to be reduced at this time of the year. The baiting operations were undertaken by the Western Australian Department of Biodiversity, Conservation and Attraction’s (DBCA) Western Shield baiting team in accordance with Permit No. PER14758 Version 2 issued by the Australian Pesticides and Veterinary Medicines Authority.

The *Eradicat*® bait is a moist ‘chipolata’ style sausage weighing ~17 g, which is injected with 4.5 mg of 1080. The bait matrix consists of 70% moist kangaroo mince, 20% chicken fat, and 10% digest and flavour enhancers [43]. Baits were trucked frozen to a nearby airstrip. On the day of baiting, baits were thawed and then sweated on racks in direct sunlight. *Coopex®* (a residual insecticide) mixed at 12.5 g/L was sprayed on the baits to reduce the likelihood of ant attack. Once ready, baits were loaded into a dedicated aircraft equipped with purpose-designed bait delivery hardware. Fifty baits were deployed at each defined drop point along flight transects 1 km apart, resulting in an application rate of 50 baits km^-2^ across the entire bait cell [27]. The bait cell for 2016 and 2017 covered 144,100 ha. It was then modified in 2018 and 2019 to incorporate potential corridors (three additional areas of favourable habitat adjacent to the former bait cell) that may have been used by feral cats to reinvade the treatment area (Fig 1). A large inaccessible portion of the rugged Hamersley Range, which was non-preferred cat habitat was excluded [44]. These changes decreased the treatment area slightly to 142,036 ha. Bait exclusion areas were placed around mine sites, public roads and waterholes along the Robe River and other major drainage lines. On average, approximately 71,500 baits were dropped in 1,430 bait clusters each year.

### Camera trapping

Due to the nature of the terrain and to enable broad coverage of both the treatment and reference sites, we set camera traps using the existing road networks. GIS mapping tools in ArcGIS were used to generate randomised camera trap sites that were within walking distance of roads (50 to 400 m either side) and ≥3 km apart. The 3 km distance was used to increase camera trap independence by reducing the chance of individual feral cats appearing on multiple cameras during the same sampling period [28]. For the treatment site at Yarraloola, camera traps were located at least 2 km inside the bait cell boundary and there was a buffer of >3 km between the bait cell boundary and the nearest camera trap on Red Hill. Sixty camera trap sites were used at each site (Fig 1). Fifty-six of the camera traps in the reference site were >10 km from the treatment site. Centroids of the two camera trap arrays (treatment and reference) were approximately 60 km apart.

Each camera (Reconyx HyperFire™ PC900, Reconyx, Wisconsin; USA) was mounted 30 cm above the ground on a 45 cm heavy duty plastic tent peg and positioned to face south. Cameras were programmed to take five pictures up to two frames per second upon a trigger, using an infra-red flash. To enhance the typically low detection rate of cats, visual and olfactory lures were used [45]. A ‘lure pole’ was set 3 m in front of each camera trap. The olfactory lure consisted of a plastic vial containing 15–20 ml of ‘Catastrophic’ scent lure in an oil suspension (Outfoxed Pest Control, Victoria), attached to a stake approximately 30 cm from the ground. Also attached to this stake, was a 1.5 m long metal curtain rod with three white turkey feathers taped obliquely at its midpoint and a 30 cm length of silver tinsel secured to the top of the rod [28]. The scent lure used to attract cats to camera traps was also found to attract northern quolls and wild dogs. Detections of wild dogs were rare, and the data were too sparse to analyse.

Vegetation was trimmed from the detection zone of the camera to minimise false triggers caused by moving plants. Camera traps were set for a minimum of 25 nights prior to the July baiting operation each year and then re-set three weeks afterwards for a further 25 nights. Camera traps and lures were removed for the intervening period to prevent cats becoming accustomed to them. All images from the camera traps were uploaded into the program ‘CPW Photo Warehouse’ [46] for processing and all fauna species (excluding cattle) identified. Date and time-stamp information from each image was captured ensuring an accurate recording of time of day for each image. The program was also used to generate detection data for cats and northern quolls for each camera trap.

Detection rate was used as an estimate of the relative activity of cats and northern quolls. We considered detections as independent when separated by greater than 15 minutes [47]. Multiple detections of northern quolls on any given night at camera trap sites in their preferred habitats were common. In contrast, it was rare for cats to be detected more than once on a camera in a single night.

### Trapping, radio-collaring and monitoring feral cats

Victor ‘Soft Catch’® No 1.5 padded leg-hold traps (Woodstream Corp., Lititz, Pa.; U.S.A.), spaced 0.5 to 1 km apart, were used to capture feral cats during trapping sessions for approximately 10 nights each in April-May 2018 and May 2019. Open-ended trap sets were used consisting of two traps positioned lengthwise and vegetation was used as a barrier along the sides of the trap area [27]. Fresh faeces from desexed domestic cats were used as the attractant. Seventy-six trap locations were set on the treatment site in 2018 and 2019. The reference site was only trapped in 2018 with 78 trap locations used.

Trapped cats were sedated with an intramuscular injection of 4 mg/kg Zoletil 100® (Virbac, Milperra, Australia), sexed, weighed, coat colour noted, and DNA tissue samples taken. Cats over 1700 g were fitted with an 80 g GPS-VHF collar (Advanced Telemetry Systems, Minnesota, USA). Collars were deployed at least two months prior to baiting and were programmed to take 24 GPS fixes per day (i.e., hourly intervals) between July–September over the baiting period, and four GPS fixes per day (i.e., 12 am, 6 am, 12 pm, 6 pm) for the remaining months. The VHF signals changed to mortality mode following 12 hours of collar inactivity.

Monitoring of radio-collared cats was conducted by ground and aerial tracking. Helicopter flights were conducted prior to and after baiting in 2018 and 2019 to locate radio-collared cats (alive or dead) and to remotely download the GPS data from each collar (download distance around 600 m). Collared cats found dead following baiting were too mummified to necropsy (>six weeks old) but there were no gross signs that scavengers had interfered with the cat carcasses. To determine if a cat was killed from bait consumption, positional data recovered from the GPS collar was reviewed against the bait drop location data in QGIS [48]. The hourly movements of the cat leading up to when the collar showed inactivity was used to determine the likelihood of intersection with a cluster of baits. The time of death following the bait drop was then estimated [28, 35].

### Statistical analysis

Changes in both feral cat and northern quoll activity were modelled using a generalised linear mixed model (GLMM) with a Poisson distribution. The response variable was the total number of detections of each species recorded over each monitoring session (i.e., 25 nights pre- and post-baiting). As some camera traps operated for less than 25 nights, we included the log of the number of nights each camera trap operated, divided by 25 nights, as an offset to account for variable sampling effort. Camera trap ID was modelled as a random intercept and fixed factors included year of monitoring (2016–2019), site (Red Hill or Yarraloola) and treatment (pre- or post-baiting) and the interaction terms. GLMMs were fitted using the ‘lme4’ package [49] in R 4.0.2 [50]. Post-hoc tests were used to examine pairwise combinations of all variables in the R package ‘emmeans’ [51]. As an indicator of model performance, we compared the full model with the intercept-only model using the second order Akaike Information Criterion (AICc) and the R package ‘MuMIn’ [52], as well as assessing model fit using the R package ‘performance’ [53].

To investigate potential range expansion of the northern quolls, we also examined the cumulative number of new camera trap sites (corrected for uneven sampling effort) at which quolls were detected with each successive 25-night monitoring session using the species accumulation curve function (random and 100 permutations) in the R package ‘vegan’ [54].

## Results

### Camera trapping

Across the four years of camera trap monitoring, there were 230 (treatment: 87; reference: 143) independent cat and 633 (treatment: 527; reference: 136) independent northern quoll detection events (S1 Table). While we use the terms pre- and post- baiting for the unbaited reference site, this is for temporal comparison only.

### Feral cat detections

**Fig 2:**
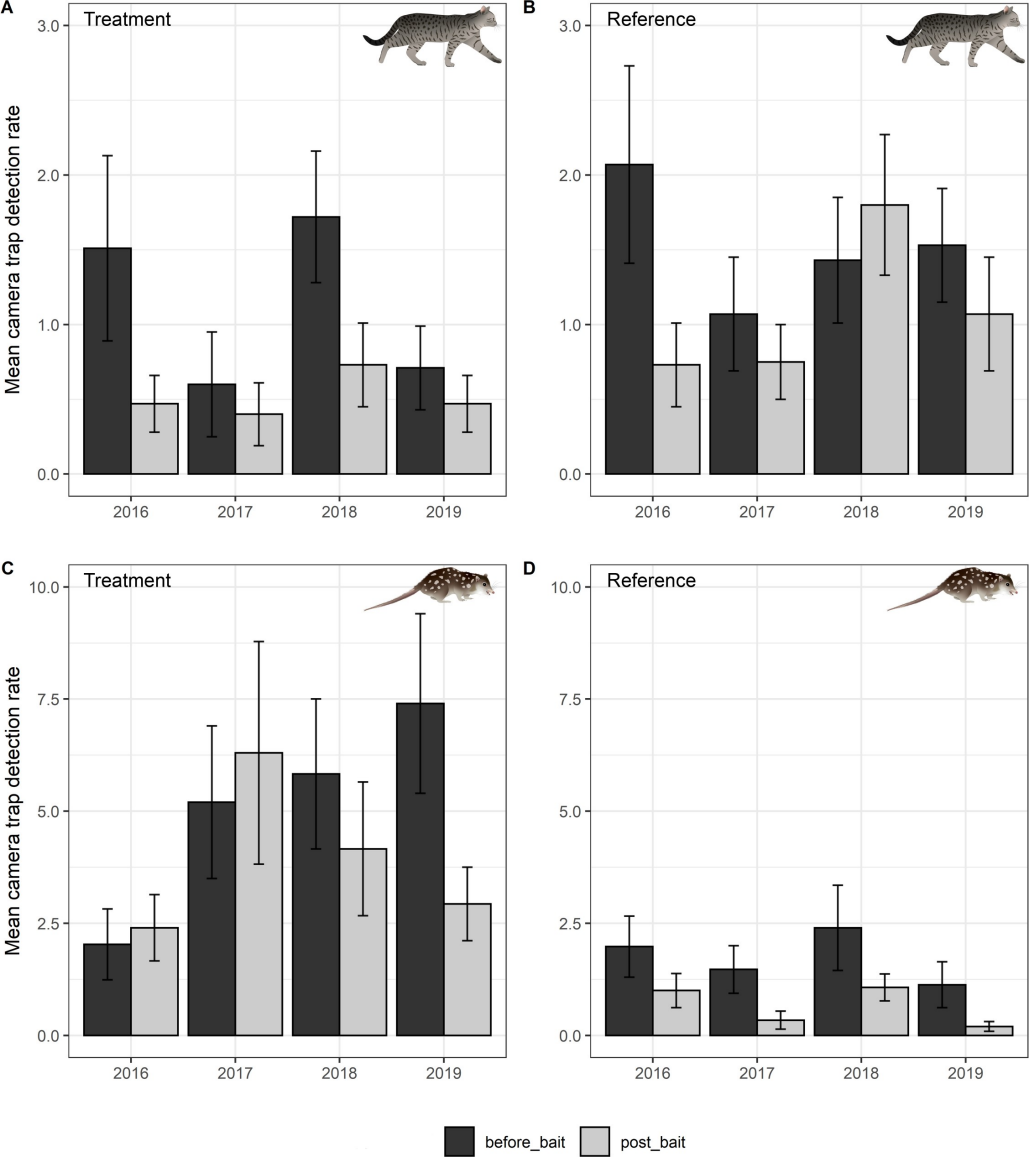
Mean detection rate (mean number of events per 100 camera trap nights per camera trap site) for feral cats (A & B) and northern quolls (C & D) at the treatment site (A & C) and reference site (B & D) prior to (before_bait) and after (post_bait) the winter baiting program from 2016 to 2019. Error bars represent standard error. Symbols courtesy of the NESP Northern Australia Hub, nespnorthern.edu.au.

The GLMM analysis for feral cat detections showed no evidence of overdispersion in the full model (Pearson Chi^2^ = 745.4; df = 941.0), the R-square of the fitted model was 0.41 and the intercept-only model performed poorly in comparison (S2 Table). The full model showed there was no significant effect of baiting on cat detections (site by treatment: *z* = 0.01, *P* = 0.998) with the change in cat detections from pre- to post-baiting being similar at the treatment and reference sites (Fig 2, S1 Fig) and this was consistent across years (S3 Table). Post hoc tests showed the only significant change in cat detections was a post-baiting decline at the reference site in 2016 (z ratio = 2.29, *P* = 0.02), and in 2018 (z-ratio = 2.3, *P* = 0.02) at the treatment site (S4 Table).

### Northern quoll detections

The GLMM analysis for northern quoll detections again showed no evidence of overdispersion (Pearson Chi^2^ = 932.7; df = 941.0), the R-square of the fitted model was 0.68 and the intercept-only model performed poorly in comparison (S2 Table). The full model showed that cat baiting had a significant effect on northern quoll detections (site by treatment: *z* = 2.19, *P* = 0.029) with the change in quoll detections from pre- to post-baiting differing between the treatment and reference sites (Fig 2, S2 Fig), which was also consistent across years (S3 Table). Post hoc tests showed a significant decline in northern quoll detections in all four years at the reference site but only in 2019 at the treatment site (S4 Table).

**Fig 3:**
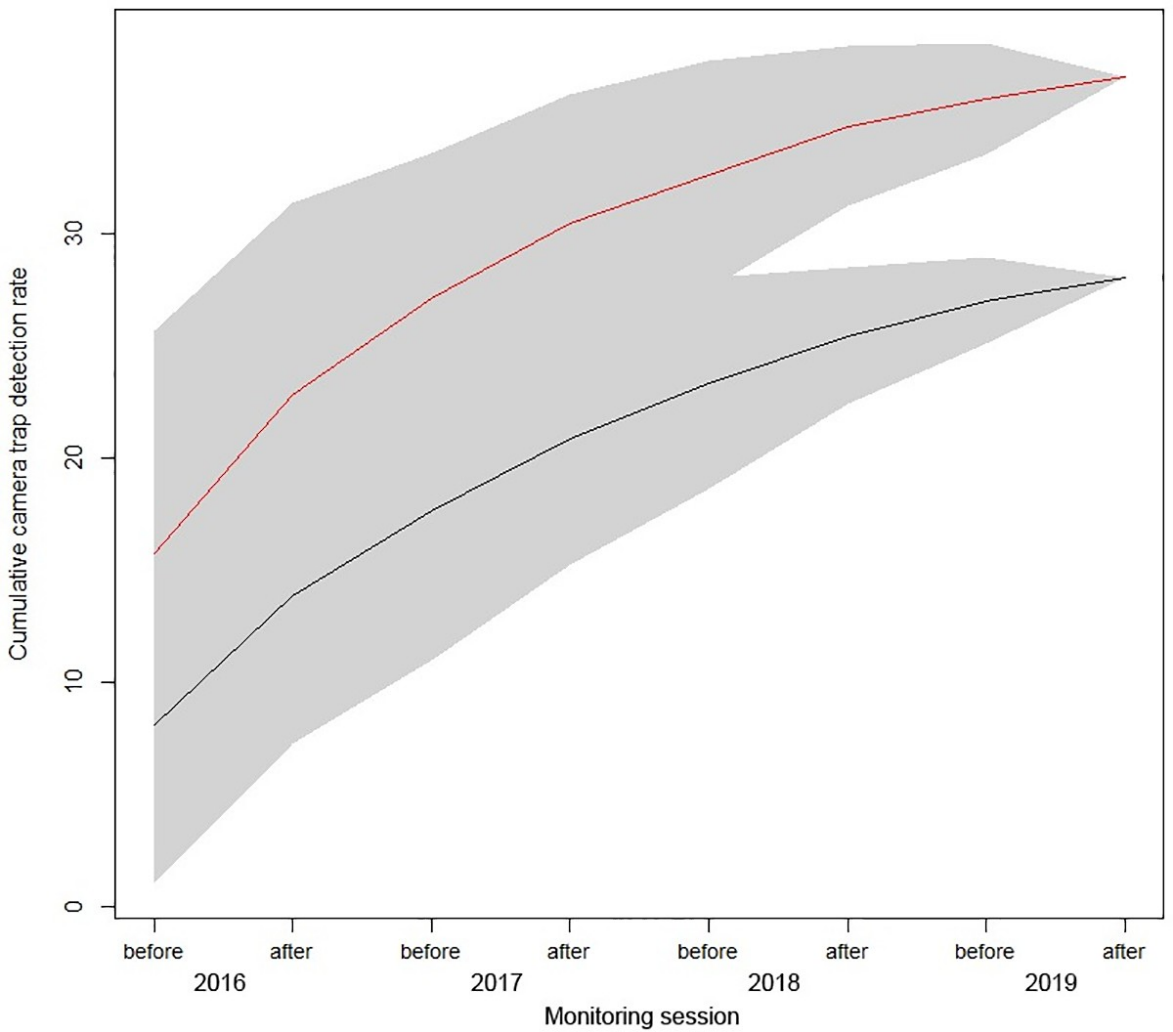
Cumulative number of camera traps on which northern quolls were detected before (b) and after (a) baiting at the treatment site (red line) and reference site (black line) from 2016 to 2019. Shaded areas represent 95% confidence intervals.

**Fig 4:**
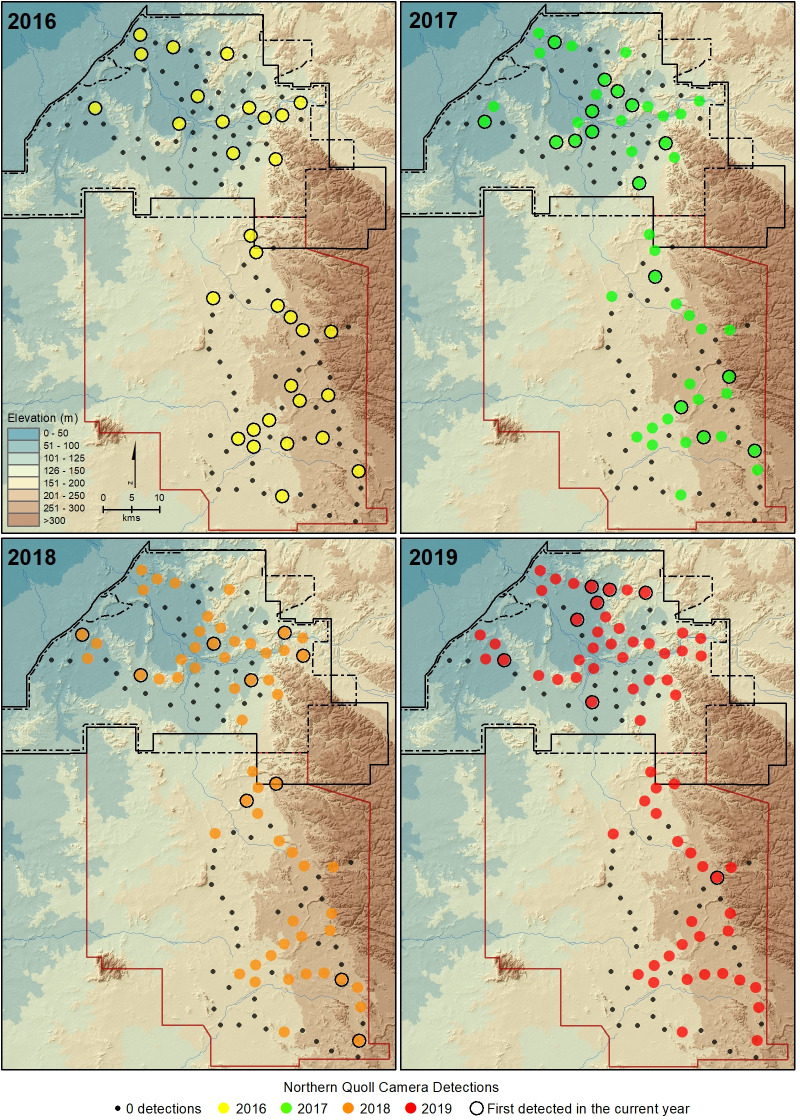
Cumulative camera trap detections of northern quolls with each successive year from 2016 to 2019 recorded within the treatment (black boundary) and reference (red boundary) sites; also showing the year in which a northern quoll was first detected at the camera trap site.

The number of new camera trap sites on which northern quolls were detected continued to rise over time at both sites (Fig 3). However, the divergence of the curves and 95% confidence intervals indicates that the cumulative increase of quoll detections on new camera trap sites was greater at the treatment site than the reference site from 2018 onwards. A spatial representation of this pattern is provided in Fig 4, which demonstrates an ongoing expansion of the spatial distribution of camera trap detections at the treatment site over time.

### Mortality of collared feral cats

Eighteen cats (11 males and seven females) were trapped and collared during the two trapping sessions at the treatment site and three (two female and one male) at the reference site in 2018 (Table 1). Most of the collared cats were adults, except for two juveniles and two sub-adults in the treatment site (S5 Table). Two cats (one sub-adult female from 2018 and one adult male from 2019) could not be located after collaring at the treatment site. Plus, another sub-adult male from 2018 died of natural causes prior to baiting in that year.

**Table 1:** Feral cats collared at the treatment and reference sites in 2018 and 2019 including the number of collared cats at each site that encountered baits and the number that died from the baiting.

Site	Year	Number of cats collared	Sex ratio (M:F)	Collars active during baiting (M:F)	Deaths attributed to baiting (M:F)	Survived exposure to baiting (M:F)	Mortality rate (%)
Treatment	2018	13 trapped	8:5	11 (7:4)	2 (1:1)	9 (6:3)	18
	2019	5 trapped	3:2	4 (2:2)	2 (1:1)	2 (1:1)	50
	2019	8 survived from 2018	5:3	8 (5:3)	2 (0:2)	6 (5:1)	25
	2019	13 total	8:5	12 (7:5)	4 (1:3)	8 (6:2)	33
Reference	2018	3 trapped	1:2	3 (1:2)	N/A	3 (1:2)	0
	2019	3 survived from 2018	1:2	3 (1:2)	N/A	3 (1:2)	0

At the treatment site, 11 collared cats were present following bait deployment in 2018, of which, two cats (one juvenile female and one adult male) were subsequently found dead with both attributed to baiting. Of the 12 cats present following bait deployment in 2019, three adult females and one adult male succumbed to baiting. There was no mortality amongst the three adult cats collared on the reference site in 2018, with all confirmed alive in September 2019 (S5 Table).

Of the five adult females exposed to bait in 2019, two of the three cats collared in 2018 died during this second exposure event and one of the two collared in 2019 died (60% mortality). None of the five adult males that survived exposure to bait in 2018 were killed during the subsequent baiting program in 2019.

## Discussion

Adoption of a broadscale aerial application of poison baiting as an operational feral cat management strategy is contingent on demonstrating both its effectiveness at removing cats from the landscape, as well as the conservation benefit it provides to native species susceptible to cat predation. As found in other studies [35, 55], proving this unequivocally can be challenging. Here, we examined the effectiveness of a landscape-scale *Eradicat®* baiting program to control feral cats, and the benefits the program provided to the threatened northern quoll, using a combination of camera traps and GPS collars fitted to cats, with mixed results.

### Efficacy of the baiting program

Despite a consistent decline in cat detections post-baiting at the treatment site each year, a similar decline was also observed at the reference site in all years except one. As such, we could not attribute this decline in cat detections to baiting. However, the mortality (18–33%) of collared cats at the treatment site after baiting indicated that the program was effective in reducing cat numbers. In a similar study and region, Comer et al. [28] demonstrated a clear effect of landscape-scale baiting using *Eradicat®* in all but one of five years of monitoring. In that year, a significant decline in site occupancy of cats was observed at both baited and unbaited sites. They suggested that a delay in the baiting program shifted the subsequent post-bait monitoring into a period when female cats were more sedentary due to nursing their young (i.e., reduced activity meant less opportunity to be detected on cameras). In a recent study in south-western Australia, Comer et al. [35] reported more variable results, with a reduction in site occupancy of cats following baiting occurring only in some years and at some sites. Their five-year study also reported variable mortality of collared cats after baiting, ranging from 0–60%. Other studies have also failed to detect a consistent response of cats to baiting [26, 43, 55–58]. Factors primarily influencing bait uptake by cats such as alternative prey availability, bait degradation/palatability, non-target bait uptake and bait shyness, were suggested as the likely reasons for this variability [55, 56, 58, 59]. For example, should a cat receive a sub-lethal dose of 1080, resulting in illness rather than death, this may lead to bait aversion and compromise the baiting program [55].

There was evidence in our study that six individually identified adult male cats (five fitted with radio-collars; one cat identified from camera trap images and then trapped and collared the following year) survived multiple baiting events [60]. Surviving adult males may have learnt to avoid baits from their previous encounters, or they had become proficient hunters of preferred live-prey and were not interested in consuming baits [26]. The long-term feral cat control program at Matuwa reported a decline in the efficacy of aerial baiting potentially as a consequence of a change in the demographics of the feral cat population towards one dominated by older and larger sized males that avoid taking baits [20]. The subsequent inclusion of a leg-hold trapping program to complement aerial baiting reversed this result by effectively re-setting the feral cat population to one that was largely bait naïve.

The average number of cat detections on camera traps at our two study sites was low (0.52–1.53 per 100 camera trap nights (CTN)) compared to some other studies. For example, in other rangeland areas of WA, Kreplins et al. [61] recorded 2.49 cat detections per 100 CTN and Doherty and Algar [57] recorded up to 4.07 cat detections per 100 CTN. The low number of cat detections for the duration of our study suggests that the density of cats within this area was generally low. Some studies have suggested that rugged rocky topography similar to our study area buffers prey populations from cat predation, as this is not the preferred habitat of feral cats [36, 44, 62, 63]. Where density is low, the power to detect change can be compromised [64]. For example, Stokeld et al. [65] reported that in tropical savanna ecosystems where cat density was also assumed to be relatively low, the sampling effort required to reliably detect cats was much higher than in temperate regions of Australia. In their study, multiple cameras at the same site dramatically increased their ability to detect cats; something to consider in future studies.

The use of attractants and various lures have been used to increase cat detection rates, with variable success [26, 45, 59, 65, 66], as well as positioning cameras in areas where encounter rates of cats are likely to be high, such as on road edges and riparian zones [58, 61, 66]. While we used both olfactory and visual lures, the camera traps were randomly placed in the landscape at between 50–400 m from roads, which may have reduced our ability to detect cats. For instance, no cats were detected at 41% of the camera trap sites used in our study. Nonetheless, the magnitude and the consistency of the decline in cat detections after baiting at the reference site was not anticipated, which highlights the importance of long-term monitoring to better understand natural variation in cat abundance over time. Feral cat populations tend to exhibit seasonal fluctuations, with summer peaks following breeding in spring, and then densities decline into winter/early spring as nutritional stress presumably takes its toll on subadult animals [67]. We sampled at a time of the year when cat densities were likely to be lowest.

However, where behavioural shifts or seasonal differences influence activity patterns, changes in activity may not reflect changes in abundance [55]. In our study, the decline in detections following the baiting in 2016, may in part be explained by reduced activity of female cats due to having dependent young [28]. The baiting operation in that year was delayed due to rain and the post-baiting monitoring was delayed until August-September. Or the efficacy of baiting could potentially be masked by survivors exploring now vacant territories left by conspecifics killed by baits [20, 68]. These individuals may be more inclined to inspect the lures at previously unvisited camera trap sites [20].

It may also be that curiosity to the lure diminishes over time and adult cats may lose interest in, or even avoid camera traps [69]. There is some evidence that animals recognise the presence of a camera and can respond either positively or negatively [70]. In this study, camera traps were likely to be easily recognisable to resident cats due to the use of tinsel as a visual lure and the repeated setting of camera traps at the same sites. For example, an adult male (RM01; S5 Table) fitted with a collar from May 2018-September 2019 at the reference site, was only detected by a camera trap on a single occasion in August 2016 (prior to collar-fitting), despite three camera trap sites being located within his home range [60]. Regular re-positioning of camera traps, or changing lures, may improve the chance of detecting camera shy individual cats.

### Effect of feral cat baiting on northern quoll detections

A precursor study to ours in the same Pilbara location demonstrated that northern quolls were unlikely to be susceptible to poison baiting using *Eradicat*® [31]. They also provided evidence of the impact of feral cats on this species with cats predating eight (20%) of the 41 radio-collared northern quolls at Yarraloola and Red Hill over a period of approximately four months. Our study supports Cowan et al. [31], by providing evidence of the positive effect of cat baiting on northern quoll activity patterns. Detection rates of northern quolls significantly declined in the post-bait monitoring session in all four years at the non-baited reference site, whereas quolls only declined by a significant level in 2019 at the baited treatment site, and slightly increased in 2016 and 2017. The significant decline in northern quoll detections at the reference site is likely to be explained by a combination of a higher level of predation by cats, decreased mating activity in the second half of August and the onset of male die-off in northern quoll populations. Monitoring prior to baiting coincided with the lead-up to mating when male northern quolls were expected to be relatively active. Post-baiting monitoring corresponded with the northern quoll’s short synchronous mating season, when males start to experience high mortality due to their poor condition [71]. The decline in northern quoll detections at the treatment site in 2019 may have been related to the extremely dry conditions experienced at this time.

According to Hernandez-Santin et al. [62], introduced predators influence the use of landscapes by northern quolls at both local and larger scales in the northern Pilbara, with quolls avoiding the flat and open habitats more frequently used by feral cats. They suggested that predator avoidance was a key reason for the contraction of the distribution of northern quolls to rocky areas across northern Australia. In our study, there was evidence that northern quolls were ranging more widely at the treatment site. This was particularly evident in the last two years of the study, with new camera detections of quolls in more open lowland habitats typically frequented by cats [44, 62, 63]. We believe the increased roaming of northern quolls is likely due to a reduction in cat predation at the treatment site. Relaxed predation pressure has been shown to directly facilitate range expansion in other meso-predators [72, 73] and there is evidence that some species utilise more open habitats when predation pressure is reduced [74, 75].

Few studies have examined the response of native prey species to landscape-scale cat baiting programs, perhaps because the typically threatened species that these programs are designed to protect are rare and cryptic and it is difficult to quantify population responses. Like our study, Comer et al. [35] reported benefits to the threatened western ground parrot (*Pezoporus flaviventris*) and chuditch (*Dasyurus geoffroii*) from a cat baiting program in southern WA, despite also finding no consistent effect of cat baiting on cat detections or site occupancy. These results suggest that the monitoring of prey populations in response to cat baiting is critical to clearly demonstrate the net benefit that landscape-scale control of feral cats provides. Three primary issues that are difficult to address in cat baiting or control programs are: (1) what proportion of the cat population needs to be removed to conserve and enhance target prey populations; (2) what are the threshold densities of cats that prey species can persist with; and (3) how frequently does long-term baiting or control need to be conducted to maintain low cat numbers in the face of reinvasion from surrounding areas? Monitoring the responses of both native prey and cat populations in the long-term would enable these questions to be addressed.

### Future directions

The development of more advanced analytical techniques for camera-trap data suggests that it is now feasible to derive robust population density estimates for cryptic and wide-ranging species based on individual identification [76, 77]. Camera traps can be placed in grid formation in a landscape to systematically sample areas of interest, then the resulting history of detections can be used to estimate the abundance of a species using a spatially explicit capture-recapture (SECR) framework. These models consider both the distribution and movement of individuals across the landscape in relation to the placement of detection devices, and account for imperfect detection [78]. Some studies have already successfully applied this approach to estimating density by individually identifying feral cats from unique coat markings [61, 63, 76, 77]. Preliminary investigations using the camera trap data captured during this study have been promising, although there appeared to be very few repeat detections of individual cats, particularly at the baited treatment site [60]. Habitat modelling based on movements of collared cats could inform the placement of camera traps by identifying high cat-traffic areas [24, 79].

A more strategic adaptive management approach that accounts for the unpredictable behaviour of feral cats may be is needed to enhance effectiveness [20, 35]. For example, a combination of aerial and targeted ground-baiting by hand may be more cost effective in the naturally fragmented rocky landscapes of the Pilbara than uniform aerial baiting across the entire focus area. Aerial application could be used to deliver baits to the lowland plains favoured by cats. Whereas ground baiting of exploration tracks in the rocky uplands may deliver more targeted cat control in an area that tends to provide refugia for native species but is less preferred by cats. Additionally, ground baiting in high density feral cat prey locations, such as along waterways, may increase exposure of feral cats to the baits. Complementing baiting with leg-hold trapping may help to remove bait-shy individuals [20]. A cost-saving option may also be to avoid baiting altogether after high-rainfall periods as prey abundance will be high and feral cats are less likely to take baits.

## Conclusion

While our study indicated that landscape-scale baiting using *Eradicat®* does not remove all cats from the target area, there was evidence of a direct knockdown of cats, and evidence of a positive effect on northern quoll populations. It is likely that the rugged rocky habitat preferred by northern quolls in the Pilbara buffered them to some extent from cat predation [36, 62, 63]. Hence, the reduction in a relatively small proportion of cats in our study was sufficient to demonstrate a benefit to northern quolls.

Our study also highlighted the importance of quantifying population responses of native prey species to a baiting program to better understand the conservation benefits it provides, and to justify the commitment of resources to the control program. Future research should aim to determine the efficacy of *Eradicat®* baiting across a range of topographies, climate zones and habitats. A more strategic approach to feral cat control by considering additional, complementary control methods, may be required to maximise the efficacy of feral cat control programs and thereby improve the conservation outlook for susceptible threatened fauna.

## Supporting information

S1 Fig Modelled camera trap detection rates of feral cats before baiting (before_bait) and post baiting (post_bait) within each year at the treatment (Yarraloola) and reference (Red Hill) sites. (TIF)

S2 Fig Modelled camera trap detection rates of northern quolls before baiting (before_bait) and post baiting (post_bait) within each year at the treatment (Yarraloola) and reference (Red Hill) sites. (TIF)

S1 Table Number of independent feral cat and northern quoll detection events (number of camera trap nights) for each year prior to and following aerial bait application at the baited treatment and unbaited reference sites. (DOCX)

S2 Table Model selection results for estimating the change in camera detection rates of feral cats and northern quolls at the treatment (baited) and reference (unbaited) sites (site), before and after baiting (treatment) and year of monitoring (year). Number of model parameters (K), maximised log-likelihood values (logLik), AICc values (AICc), AICc differences (ΔAICc) and Akaike weights are shown.
(DOCX)

S3 Table Output from the generalised linear mixed model analyses for detections of feral cats and northern quolls at the treatment (Yarraloola) and reference (Red Hill) sites (site), before and after baiting (treatment) and year of monitoring (year). Standard errors (SE) and upper (UCL) and lower (LCL) 95% confidence intervals (CI) are also shown. Both models included camera ID as a random intercept.
(DOCX)

S4 Table Post-hoc Tukey tests of the difference between pre- and post-baiting detections of feral cats and northern quolls at the treatment and reference sites in each of the four years of the study. Standard Error (SE) and 95% lower (LCL) and upper (UCL) confidence intervals are also shown.
(DOCX)

S5 Table Details of feral cats captured for collaring and their fate following the winter baiting programs in 2018 and 2019. (DOCX)
