# Supplementary figures and images for: Does aerial baiting for controlling feral cats in a heterogeneous landscape confer benefits to a threatened native meso-predator?
Source: PLoS One. 2021 May 7;16(5):e0251304. doi: 10.1371/journal.pone.0251304 (PMC8104397; doi:10.1371/journal.pone.0251304)

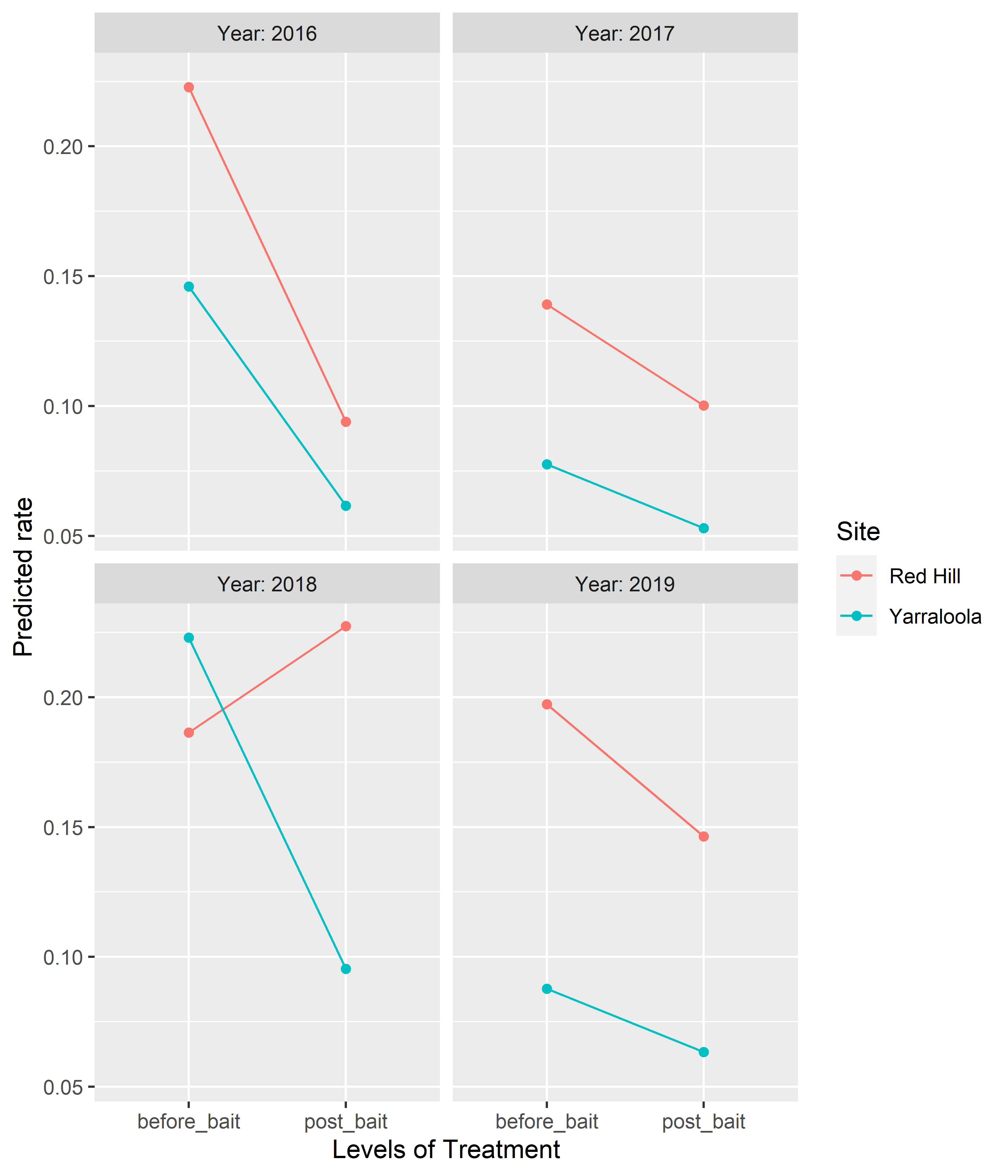

Supplement: S1 Fig — (TIF) [file pone.0251304.s001.tif]

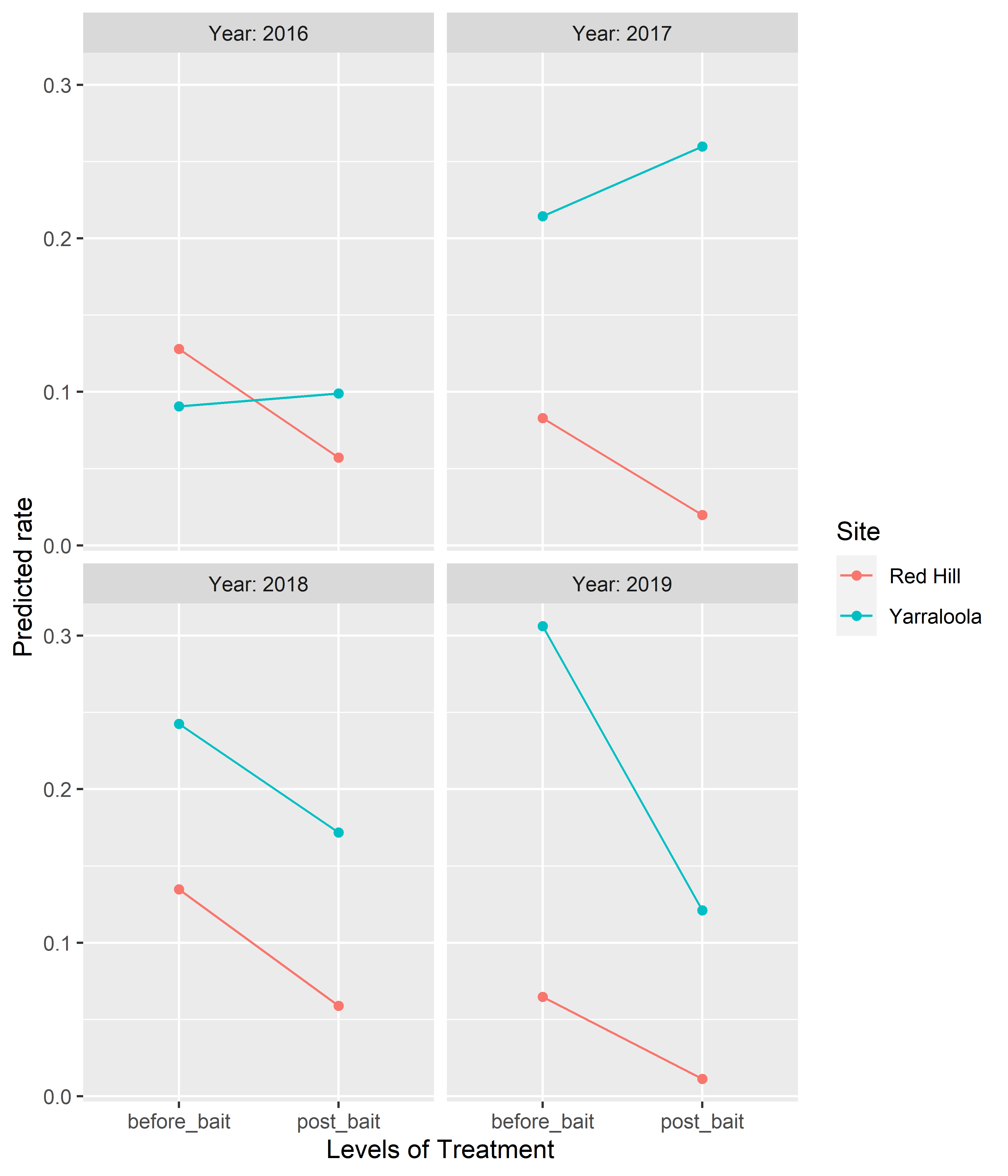

Supplement: S2 Fig — (TIF) [file pone.0251304.s002.tif]
