# Supplementary material for: Does aerial baiting for controlling feral cats in a heterogeneous landscape confer benefits to a threatened native meso-predator?
Source: PLoS One. 2021 May 7;16(5):e0251304. doi: 10.1371/journal.pone.0251304 (PMC8104397; doi:10.1371/journal.pone.0251304)
Supplement: S1 Table — (DOCX) [file pone.0251304.s003.docx]

**S1 Table.** Number of independent feral cat and northern quoll detection events (number of camera trap-nights) for each year prior to (pre-bait) and following (post-bait) aerial bait application at the baited treatment and unbaited reference sites.

| **Species** | **Year** | **Treatment**  **baited** | | **Reference**  **unbaited** | |
| --- | --- | --- | --- | --- | --- |
|  |  | **pre-bait** | **post-bait** | **pre-bait** | **post-bait** |
| Feral cat | 2016 | 15 (1313) | 7 (1485) | 20 (1267) | 11 (1472) |
|  | 2017 | 9 (1500) | 6 (1469) | 16 (1431) | 11 (1412) |
|  | 2018 | 22 (1270) | 11 (1485) | 19 (1278) | 27 (1495) |
|  | 2019 | 10 (1474) | 7 (1472) | 23 (1431) | 16 (1434) |
| Northern quoll | 2016 | 30 (1313) | 36 (1485) | 26 (1267) | 15 (1472) |
|  | 2017 | 78 (1500) | 94 (1469) | 22 (1431) | 5 (1412) |
|  | 2018 | 72 (1270) | 62 (1485) | 32 (1278) | 16 (1495) |
|  | 2019 | 111 (1474) | 44 (1472) | 17 (1431) | 3 (1434) |
