# Supplementary material for: Does aerial baiting for controlling feral cats in a heterogeneous landscape confer benefits to a threatened native meso-predator?
Source: PLoS One. 2021 May 7;16(5):e0251304. doi: 10.1371/journal.pone.0251304 (PMC8104397; doi:10.1371/journal.pone.0251304)
Supplement: S2 Table — Number of model parameters (K), maximised log-likelihood values (logLik), AICc values (AICc), AICc differences (ΔAICc) and Akaike weights are shown. (DOCX) [file pone.0251304.s004.docx]

**S2 Table.** Model selection results for estimating the change in camera detection rates of feral cats and northern quolls at the treatment (baited) and reference (unbaited) sites (site), before and after baiting (treatment) and year of monitoring (year). Number of model parameters (K), maximised log-likelihood values (logLik), AICc values (AICc), AICc differences (ΔAICc) and Akaike weights are shown.

| **Species** | **Model*** | **K** | **logLik** | **AICc** | **ΔAICc** | **AICc weight** |
| --- | --- | --- | --- | --- | --- | --- |
| Feral cat | site x treatment x year | 17 | -364.57 | 763.80 | 0.00 | 0.99 |
|  | intercept-only | 2 | -385.04 | 774.10 | 10.29 | 0.01 |
| Northern quoll | site x treatment x year | 17 | -558.51 | 1151.70 | 0.00 | 1.00 |
|  | intercept-only | 2 | -637.61 | 1279.20 | 127.57 | 0.00 |

*All models include camera trap ID as a random intercept
