# Supplementary material for: Does aerial baiting for controlling feral cats in a heterogeneous landscape confer benefits to a threatened native meso-predator?
Source: PLoS One. 2021 May 7;16(5):e0251304. doi: 10.1371/journal.pone.0251304 (PMC8104397; doi:10.1371/journal.pone.0251304)
Supplement: S3 Table — Standard errors (SE) and upper (UCL) and lower (LCL) 95% confidence intervals (CI) are also shown. Both models included camera ID as a random intercept. (DOCX) [file pone.0251304.s005.docx]

**S3 Table.** Output from the generalised linear mixed model analyses for detections of feral cats and northern quolls at the treatment and reference sites (Site), before and after baiting (Treatment) and year of monitoring (Year). Standard errors (SE), lower (LCL) and upper (UCL) 95% confidence intervals are also shown. Both models included camera ID as a random intercept.

| **Species** | **Model term** | **Estimate** | **SE** | **LCL** | **UCL** | **z value** | ***P*** |
| --- | --- | --- | --- | --- | --- | --- | --- |
| Feral cat | (Intercept) | -1.411 | 0.288 | -1.977 | -0.846 | -4.894 | <0.001 |
|  | Year2017 | -0.471 | 0.337 | -1.131 | 0.190 | -1.397 | 0.163 |
|  | Year2018 | -0.179 | 0.322 | -0.811 | 0.453 | -0.554 | 0.580 |
|  | Year2019 | -0.122 | 0.308 | -0.724 | 0.481 | -0.396 | 0.692 |
|  | SiteYarraloola | -0.423 | 0.417 | -1.239 | 0.394 | -1.015 | 0.310 |
|  | Treatmentpost_bait | -0.863 | 0.377 | -1.602 | -0.125 | -2.291 | 0.022 |
|  | Year2017:SiteYarraloola | -0.161 | 0.541 | -1.221 | 0.898 | -0.299 | 0.765 |
|  | Year2018:SiteYarraloola | 0.602 | 0.466 | -0.311 | 1.515 | 1.292 | 0.196 |
|  | Year2019:SiteYarraloola | -0.388 | 0.512 | -1.391 | 0.616 | -0.758 | 0.449 |
|  | Year2017:Treatmentpost_bait | 0.535 | 0.544 | -0.531 | 1.601 | 0.983 | 0.326 |
|  | Year2018:Treatmentpost_bait | 1.062 | 0.482 | 0.118 | 2.006 | 2.205 | 0.028 |
|  | Year2019:Treatmentpost_bait | 0.565 | 0.499 | -0.413 | 1.543 | 1.132 | 0.258 |
|  | SiteYarraloola:Treatmentpost_bait | 0.002 | 0.594 | -1.162 | 1.166 | 0.003 | 0.998 |
|  | Year2017:SiteYarraloola:Treatmentpost_bait | -0.054 | 0.886 | -1.790 | 1.682 | -0.061 | 0.952 |
|  | Year2018:SiteYarraloola:Treatmentpost_bait | -1.049 | 0.761 | -2.541 | 0.442 | -1.379 | 0.168 |
|  | Year2019:SiteYarraloola:Treatmentpost_bait | -0.029 | 0.838 | -1.671 | 1.614 | -0.034 | 0.973 |
| Northern quoll | (Intercept) | -1.965 | 0.388 | -2.725 | -1.206 | -5.070 | <0.001 |
|  | Year2017 | -0.435 | 0.292 | -1.008 | 0.139 | -1.486 | 0.137 |
|  | Year2018 | 0.050 | 0.268 | -0.474 | 0.575 | 0.189 | 0.850 |
|  | Year2019 | -0.681 | 0.315 | -1.298 | -0.064 | -2.163 | 0.031 |
|  | SiteYarraloola | -0.346 | 0.501 | -1.328 | 0.636 | -0.690 | 0.490 |
|  | Treatmentpost_bait | -0.807 | 0.327 | -1.447 | -0.167 | -2.470 | 0.014 |
|  | Year2017:SiteYarraloola | 1.296 | 0.363 | 0.584 | 2.008 | 3.569 | <0.001 |
|  | Year2018:SiteYarraloola | 0.934 | 0.345 | 0.258 | 1.611 | 2.707 | 0.007 |
|  | Year2019:SiteYarraloola | 1.899 | 0.376 | 1.161 | 2.636 | 5.043 | <0.001 |
|  | Year2017:Treatmentpost_bait | -0.626 | 0.594 | -1.790 | 0.537 | -1.055 | 0.291 |
|  | Year2018:Treatmentpost_bait | -0.021 | 0.448 | -0.899 | 0.857 | -0.047 | 0.962 |
|  | Year2019:Treatmentpost_bait | -0.940 | 0.707 | -2.325 | 0.445 | -1.330 | 0.183 |
|  | SiteYarraloola:Treatmentpost_bait | 0.896 | 0.410 | 0.092 | 1.699 | 2.185 | 0.029 |
|  | Year2017:SiteYarraloola:Treatmentpost_bait | 0.730 | 0.661 | -0.566 | 2.026 | 1.104 | 0.270 |
|  | Year2018:SiteYarraloola:Treatmentpost_bait | -0.413 | 0.541 | -1.473 | 0.646 | -0.764 | 0.445 |
|  | Year2019:SiteYarraloola:Treatmentpost_bait | -0.076 | 0.770 | -1.585 | 1.433 | -0.099 | 0.921 |
