# Supplementary material for: Does aerial baiting for controlling feral cats in a heterogeneous landscape confer benefits to a threatened native meso-predator?
Source: PLoS One. 2021 May 7;16(5):e0251304. doi: 10.1371/journal.pone.0251304 (PMC8104397; doi:10.1371/journal.pone.0251304)
Supplement: S4 Table — Standard Error (SE) and 95% lower (LCL) and upper (UCL) confidence intervals are also shown. (DOCX) [file pone.0251304.s006.docx]

**S4 Table.** Post-hoc Tukey tests of the difference between pre- (before_bait) and post-baiting (post_bait) detections of feral cats and northern quolls at the treatment and reference sites in each of the four years of the study. Standard Error (SE) and 95% lower (LCL) and upper (UCL) confidence intervals are also shown.

| **Species** | **Site** | **Year** | **Treatment** | **Detection rate** | **SE** | **LCL** | **UCL** | ***P*** |
| --- | --- | --- | --- | --- | --- | --- | --- | --- |
| Feral cat | Reference | 2016 | before_bait | 0.223 | 0.064 | 0.127 | 0.392 | 0.022 |
|  |  |  | post_bait | 0.094 | 0.033 | 0.047 | 0.188 |  |
|  |  | 2017 | before_bait | 0.139 | 0.043 | 0.075 | 0.257 | 0.403 |
|  |  |  | post_bait | 0.100 | 0.035 | 0.050 | 0.201 |  |
|  |  | 2018 | before_bait | 0.186 | 0.055 | 0.104 | 0.333 | 0.508 |
|  |  |  | post_bait | 0.227 | 0.061 | 0.135 | 0.384 |  |
|  |  | 2019 | before_bait | 0.197 | 0.055 | 0.114 | 0.342 | 0.360 |
|  |  |  | post_bait | 0.146 | 0.046 | 0.080 | 0.269 |  |
|  | Treatment | 2016 | before_bait | 0.146 | 0.047 | 0.077 | 0.275 | 0.060 |
|  |  |  | post_bait | 0.062 | 0.026 | 0.027 | 0.141 |  |
|  |  | 2017 | before_bait | 0.078 | 0.030 | 0.037 | 0.165 | 0.470 |
|  |  |  | post_bait | 0.053 | 0.024 | 0.022 | 0.128 |  |
|  |  | 2018 | before_bait | 0.223 | 0.064 | 0.127 | 0.391 | 0.022 |
|  |  |  | post_bait | 0.095 | 0.034 | 0.047 | 0.192 |  |
|  |  | 2019 | before_bait | 0.088 | 0.032 | 0.043 | 0.181 | 0.510 |
|  |  |  | post_bait | 0.063 | 0.027 | 0.028 | 0.145 |  |
| Northern quoll | Reference | 2016 | before_bait | 0.128 | 0.050 | 0.060 | 0.274 | 0.014 |
|  |  |  | post_bait | 0.057 | 0.024 | 0.025 | 0.131 |  |
|  |  | 2017 | before_bait | 0.083 | 0.033 | 0.038 | 0.181 | 0.004 |
|  |  |  | post_bait | 0.020 | 0.011 | 0.007 | 0.059 |  |
|  |  | 2018 | before_bait | 0.135 | 0.051 | 0.064 | 0.284 | 0.007 |
|  |  |  | post_bait | 0.059 | 0.025 | 0.026 | 0.134 |  |
|  |  | 2019 | before_bait | 0.065 | 0.027 | 0.029 | 0.146 | 0.005 |
|  |  |  | post_bait | 0.011 | 0.008 | 0.003 | 0.042 |  |
|  | Treatment | 2016 | before_bait | 0.091 | 0.032 | 0.045 | 0.183 | 0.720 |
|  |  |  | post_bait | 0.099 | 0.035 | 0.050 | 0.196 |  |
|  |  | 2017 | before_bait | 0.214 | 0.070 | 0.113 | 0.407 | 0.210 |
|  |  |  | post_bait | 0.260 | 0.084 | 0.138 | 0.490 |  |
|  |  | 2018 | before_bait | 0.243 | 0.080 | 0.128 | 0.462 | 0.046 |
|  |  |  | post_bait | 0.172 | 0.057 | 0.090 | 0.329 |  |
|  |  | 2019 | before_bait | 0.306 | 0.098 | 0.163 | 0.574 | <0.001 |
|  |  |  | post_bait | 0.121 | 0.041 | 0.062 | 0.237 |  |
