# Supplementary material for: Does aerial baiting for controlling feral cats in a heterogeneous landscape confer benefits to a threatened native meso-predator?
Source: PLoS One. 2021 May 7;16(5):e0251304. doi: 10.1371/journal.pone.0251304 (PMC8104397; doi:10.1371/journal.pone.0251304)
Supplement: S5 Table — (DOCX) [file pone.0251304.s007.docx]

**S5 Table.** Details of feral cats captured at the treatment and reference sites for collaring and their fate following the winter baiting programs in 2018 and 2019.

| **Cat code** | **Capture date** | **Sex** | **Mass (g)** | **Coat colour** | **Age*** | **Status** |
| --- | --- | --- | --- | --- | --- | --- |
| **Treatment site – Aerial baiting 9-10 July 2018** | | | |  |  |  |
| YF01 | 25-Apr-18 | ♀ | 2400 | Tabby | Sub-adult | Not relocated since collared |
| YF02 | 26-Apr-18 | ♀ | 1700 | Tabby | Juvenile | **Died from baiting** (0300 hrs 12/07/18) |
| YF03 | 26-Apr-18 | ♀ | 3390 | Tabby | Adult | Survived |
| YM02 | 27-Apr-18 | ♂ | 4170 | Tabby | Adult | Survived |
| YM03 | 28-Apr-18 | ♂ | 4200 | Tabby | Adult | Survived |
| YM04 | 28-Apr-18 | ♂ | 5000 | Tabby | Adult | Survived**; Died (**9/09/18) natural causes |
| YF04 | 28-Apr-18 | ♀ | 3120 | Tabby | Adult | Survived |
| YF05 | 30-Apr-18 | ♀ | 2630 | Tortoiseshell | Adult | Survived |
| YM05 | 1-May-18 | ♂ | 2050 | Tabby | Juvenile | **Died** prior to baiting (28/06/18) natural causes |
| YM06 | 1-May-18 | ♂ | 4630 | Black | Adult | Survived |
| YM07 | 1-May-18 | ♂ | 5620 | Tabby | Adult | Survived |
| YM08 | 2-May-18 | ♂ | 3080 | Tabby | Sub-adult | Survived |
| YM09 | 4-May-18 | ♂ | 4400 | Tabby | Adult | **Died from baiting** (0800 hrs 28/07/18) |
| **Treatment site – Aerial baiting 8-9 July 2019** | | | |  |  |  |
| YF03 | Previous year | ♀ |  | Tabby | Adult | **Died from baiting** (1300 hrs 26/07/19) |
| YM02 | Previous year | ♂ |  | Tabby | Adult | Survived |
| YM03 | Previous year | ♂ |  | Tabby | Adult | Survived |
| YF04 | Previous year | ♀ |  | Tabby | Adult | **Died from baiting** (0800 hrs 16/07/19) |
| YF05 | Previous year | ♀ |  | Tortoiseshell | Adult | Survived |
| YM06 | Previous year | ♂ |  | Black | Adult | Survived |
| YM07 | Previous year | ♂ |  | Tabby | Adult | Survived |
| YM08 | Previous year | ♂ |  | Tabby | Adult | Survived |
| YF06 | 23-May-19 | ♀ | 2930 | Tabby | Adult | **Died from baiting** (1300 hrs, 11/07/19) |
| YF07 | 28-May-19 | ♀ | 4040 | Tabby | Adult | Survived |
| YM10 | 25-May-19 | ♂ | 4075 | Tabby | Adult | **Died from baiting** (1000 hrs, 16/07/19) |
| YM11 | 28-May-19 | ♂ | 4535 | Black | Adult | Survived |
| YM12 | 29-May-19 | ♂ | 4190 | Black | Adult | Not relocated since collared |
| **Reference site – 2018 and 2019 (no baiting)** | | | |  |  |  |
| RM01 | 13-May-18 | ♂ | 5100 | Tabby | Adult | Alive on 10/09/19 |
| RF01 | 14-May-18 | ♀ | 3000 | Tabby | Adult | Alive on 10/09/19 |
| RF02 | 16-May-18 | ♀ | 2900 | Tabby | Adult | Alive on 4/09/19 |

* from: Jones E, Coman BJ. Ecology of the feral cat, Felis catus (L.), in south-eastern Australia II. Reproduction. Wildlife Research. 1982;9(1): 111-119. (adults: males >3.5 kg and females >2.4 kg; sub-adults: males 2.2-3.5 kg and females 1.9-2.4 kg; juveniles: males <2.2 kg and females <1.9 kg)
